# In vitro atomization analysis and evaluation of inhalable sodium sivelestat formulations

**DOI:** 10.1371/journal.pone.0309721

**Published:** 2024-09-20

**Authors:** Rangdong Liu, Aifang He, Yan Xu, Yisheng Zhou, Hui Cao

**Affiliations:** 1 Zhuhai College of Science and Technology, Zhuhai, China; 2 Jinan University, College of Pharmcy, Guangzhou, China; 3 Increase Pharma (Hengqin) Institute Co. LTD, Zhuhai, China; University of Helsinki, FINLAND

## Abstract

The purpose of this paper was to study in vitro atomization properties of the self-developed sodium sivelestat for inhalation, evaluate the feasibility of this preparation as an aerosol inhalation, and provide the guidance for the following animal administration experiment. Firstly, in order to ensure accurate, uniform and stable doses of the self-developed product after administration, its atomization performance was analyzed through the testing of fine particle mass and the total emitted dose, and the results of its atomization parameters meet the requirement of inhalation. Next, Atomization characteristics of two commonly used nebulizers, air compressed nebulizer and mesh nebulizer, were studied and compared. The results showed that mesh atomizers have a smaller and more uniform particle size distribution. And then, the experiment of acute lung injury induced by aerosol inhalation of lipopolysaccharide in mice was used to test the therapeutic effect of our self-developed formulation, and compared with the positive control (sodium sivelestat for injection). The results showed that inhalation had a lower concentration and was equally effective than injection of sodium sivelestat. All the results support that the self-developed sodium sivelestat can be used as an aerosol inhaled drug.

## Introduction

Acute lung injury/acute respiratory distress syndrome is a prevalent critical illness characterized by a high mortality, significantly endangering the lives of severe patients and impeding their quality of life [1, 2]. This condition often leads to an imbalance between pro-inflammatory and anti-inflammatory responses within the body, resulting in compromised immune function and sustained uncontrolled systemic inflammatory response syndrome [3–6]. Sodium sivelestat for injection is a commonly employed treatment for acute lung damage associated with systemic inflammatory response syndrome. However, it frequently leads to abnormal liver function, manifesting as elevated levels of aspartate aminotransferase, alanine aminotransferase, alkaline phosphatase, and bilirubin, as a common side effect of systemic administration [7].

Pulmonary inhalation therapy is widely applied in the treatment of pulmonary diseases, including pneumonia, asthma, and chronic obstructive pulmonary disease [8, 9]. This mode of administration allows medications to directly reach the affected site, offering distinct advantages over other delivery methods. Leveraging the lung’s physiological characteristics, such as its thin alveolar epithelium, abundant pulmonary capillaries, and extensive absorption area, pulmonary inhalation formulations can promptly target the lesion and take effect rapidly. Additionally, this approach bypasses the liver’s first-pass effect, enhancing the drug’s bioavailability. It also permits lower dosages and reduces the risk of toxic side effects. Consequently, pulmonary inhalation administration is considered the most favorable route for treating localized lung lesions [10, 11]. Although the injection of sivelestat sodium, which is administered through atomization, is flawed, these studies have shown that pulmonary exposure to inhaled sodium sivelastat administered as an intratracheal bolus showed no discrepancy compared with systemic administration via the injection [12, 13].

These findings led us to realize the feasibility of developing sodium sivelestat into inhaled formulations. Considering the chemical instability of sodium sivelestat [14], we tried to develop it into freeze-dried powder for atomizing inhalation. Subsequently, the inhalable formulation was subjected to evaluation from three key perspectives: delivery rate and total emitted dose, aerodynamic particle size distribution, and spray modes [15–17]. Hereon, atomization characteristics of two commonly used nebulizers, air compressed nebulizer and mesh nebulizer, were studied and compared. Furthermore, we conducted a preliminary assessment of the therapeutic efficacy of our self-developed formulation using an experiment involving acute lung injury induced by aerosol inhalation of lipopolysaccharide in mice [18, 19]. This allowed us to gain initial insights into the potential benefits and effectiveness of our formulation [20–22].

## Materials and methods

### Chemical reagents and pharmacodynamic samples

Sodium sivelestat for inhalation was developed by our group (Increasepharma (Hengqin) Institute Co. LTD). Sodium sivelestat for injection was purchased from Suzhou Erye Pharmaceutical Co. LTD. Sivelestat (97.40%) and sodium propyl p-hydroxybenzoate (98.28%), absolute ethyl alcohol, sodium dihydroxyphosphate, potassium dihydroxyphosphate, sodium chloride and sodium bicarbonate were purchased from Sinopharmachem. Acetonitrile was obtained from OCEANPAK. Tween 80 was obtained from Xian Jinxiang Pharmaceutical excipients Co., LTD. Water was deionized. Lipopolysaccharide (LPS) were purchased from Biotopped. Normal saline were purchased from Shijiazhuang four pharmaceutical Co. LTD. Hepes was purchased from solarbio. Dimethyl sulfoxide was purchased from Beijing Bairdi Biotechnology Co. LTD. Brij and Elastase from porcine pancreas were purchased from sigma-aldrich. MeOSuc-Ala-Pro-Met-Pna was purchased from Nanjing peptide industry Biotechnology Co. LTD. Phosphate buffer salt was purchased from solarbio. Mouse α1-AT ELISA Kit was purchased from Shanghai Jianglai Biotechnology Co. LTD. Mouse IL-6 ELISA Kit and Mouse TNF-α ELISA Kit were purchased from solarbio. ICR mice (SPF grade) was purchased from Beijing Vitonglihua Experimental Animal Technology Co. LTD.

### Instrumentation

SB-1637 next generation impaction (NGI), BRS2100 breath simulator, LCP-5 pump, TPK-2100 flow controller, DFM2000 electronic flowmeter, cooling instruction, BRS filter membrane, and Micro-orifice collector (MOC) filter membrane were obtained from Copley Co. LTD (England). Master-s15 pure machine was purchased from Shanghai Hetai Instrument Co. LTD (China). Particle size analyser was purchased TSI Incorporated (America, mode: 3321). The HPLC system consisted of an agilent 1260 HPLC pump and a DAD detector (America). Data acquisition and processing were performed with an agilent EZ chrome chromatography data system (America). 403H compressed air nebulizer (A) and 085 compressed air nebulizer (B) were purchased from Yuwell (China) and PARI (German), respectively. Air Pro Ⅷ Mesh nebulizer (C) and type M105 Mesh nebulizer(D) were obtained from Feellife (China) and Yuwell (China), respectively. The parameters of each atomizer used in this paper are shown in S1 Table. Type A2 biological safety cabinet was purchased from Thermo (America). Individually Ventilated Cages (IVC) cage was purchased from Shenzhen Hongteng Biotechnology Co. LTD (China). Animal oral-nasal inhalation exposure system was purchased from Shanghai Meili Experimental Technology Co. LTD (China). High volume tissue grinder was purchased from Ningbo Xinzhi Co. LTD (China). Table top high speed refrigerated centrifuge was purchased from Eppendorf (German). Multimode reader was purchased from Bio-Tek (America). Microplate incubator was purchased from Hangzhou Aosheng Instrument Co. LTD (China).

### Preparation of a lyophilized powder formulation of sodium sivelestat for inhalation

The formulation consists of sodium sivelestat tetrahydrate, Tween 80, anhydrous ethanol, phosphate buffer salt, and sodium chloride. 1.5 g of sivelestat sodium tetrahydrate was dissolved in 70 mL of distilled water, and then 0.02 g of Tween 80 was added to this solution. Next, the pH of this solution was adjusted to 7.8 using 0.1 M phosphate buffer salt (pH 12.0). Lastly, the osmotic pressure was adjusted by adding 0.46 g sodium chloride and distilled water to 100 mL mark, stirring the solution until it becomes clear. Then, 30 mL of anhydrous ethanol was added to this solution and mixed it thoroughly. It was filtered aseptically throught a 0.22 *μ*m filter into the 20 mL penicillin vial (5 mL/vial), and freeze-dried. The freeze-drying process is divided into three stages, the pre-freezing stage reduced the temperature to -45°C within 1 hour, and maintained this temperature for 4 hours; In the first drying stage, the pressure was reduced to about 2 bar using a mechanical pump. Meanwhile, the temperature was increased to -30°Cat 5°C/h. Then, the temperature was raised to -20°Cat 1°C/h. Finally, the temperature was increased to -6°Cat 6°C/h, and maintained it for 7 h. In the secondary drying stage, the temperature wass raised from -6°C to 40°C within 6 h and maintained it at 40°C for 5 hours.

### Droplet size analysis

**Fig 1:**
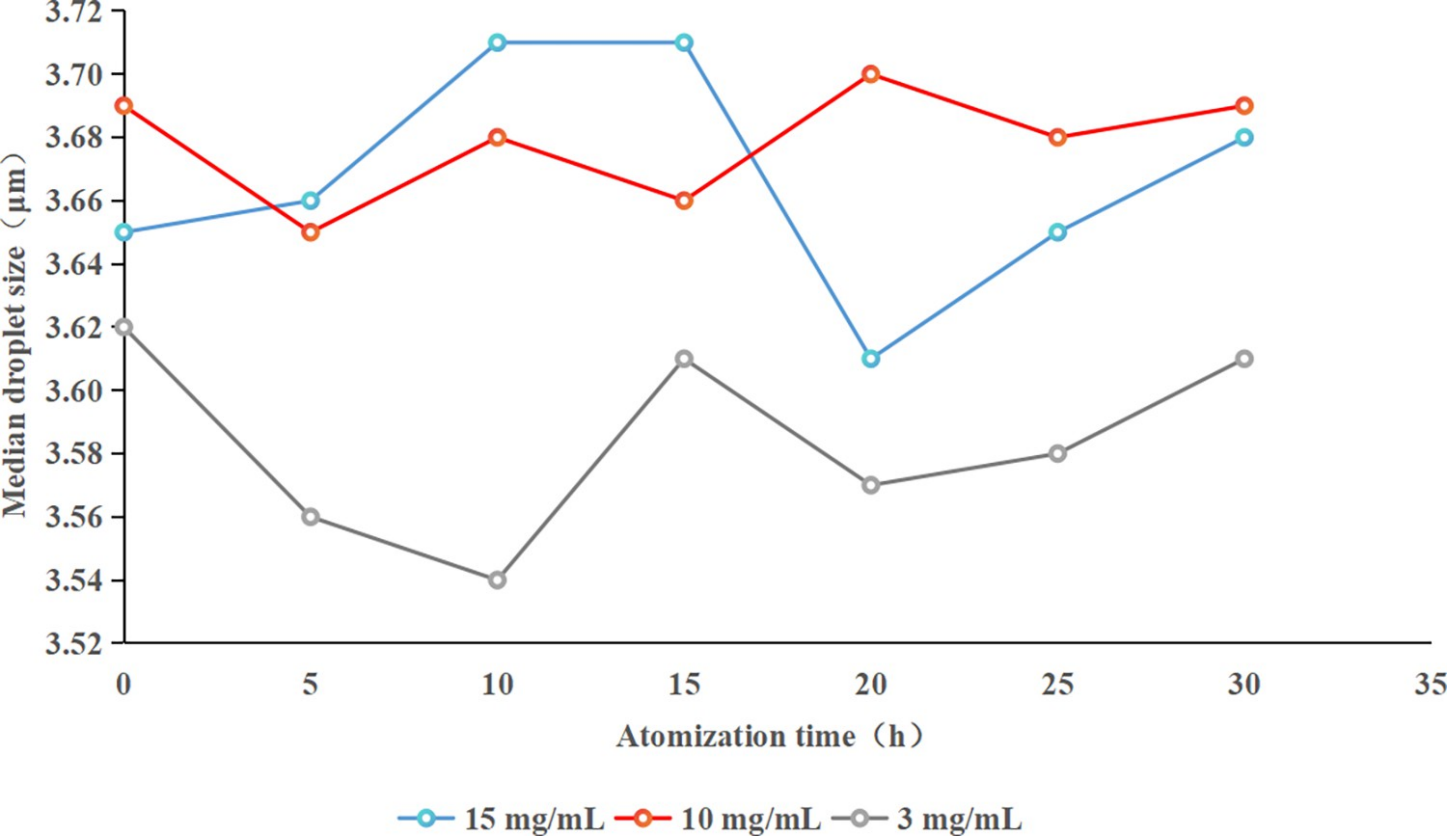
The droplet size graph of self-developed formulation.

Using PARI blue filter atomizer and TSI particle size detector, the detection principle is two-beam laser particle size measurement. The theoretical value of aerosol droplet size (<5 *μ*m) was obtained by adjusting the flow rate of atomizer 7.5 L/min. Finally, it is adjusted according to the actual droplet size of the drug. Self-developed formulation solution of 15 mg/mL, 10 mg/mL and 3 mg/mL was added to the atomizer respectively. In order to stabilize the aerosol droplet size, the aerosol droplet size at the spray outlet was detected after 10 minutes of atomization time. At the same time, three detection ports are selected in parallel. Droplet size values at each outlet at different time intervals was measured, including 0 min, 5 min, 10 min, 15 min, 20 min, 25 min, and 30 min. And the concentration difference of tested aerosols was determined continuously, which should not exceed ±20% of the mean value to be considered as stable. The measuring results are shown in Fig 1.

### Measurement of fine particle mass

The four nebulizer A/B and C/D were divided into two types with nebulizing time for 1, 2, 3, 4 and 5 min respectively. After 4 or 5 min of nebulizing, NGI collection surfacs at all levels were seen visually. After 3 min of nebulization, the nebulized droplets on the collecting plate are deformed obviously, and there are connected lines of droplets on the collecting plate, indicating overload phenomenon. For the nebulizer A and B, when the nebulization time was 1 min, the deposition dose was small, which would enlarge the measurement error. Therefore, the nebulization time can be set to 2 min. For the nebulizer C and D, nebulizering 2 or 3 min is not enough due to the low deposition amount, but 5 min would result in overload. Obviously, the nebulization time can be set to 4 min.

According to the four general Rules 0951 of the 2020 edition of Chinese Pharmacopoeia [15], the NGI is placed at least 90 minutes in the condition of 5°C±3°C in advance, then the vacuum pump is started and the flow rate is adjusted to 15 L/min (±5%), and the flowmeter is removed. Accurately measure 2 mL of 10 mg/mL self-developed formulation solution into the nebulization cup, connect the nebulizer to the artificial throat through the interface in the direction of use, open the nebulizer and flow control valve at the same time, close the nebulizer after nebulization, and close the vacuum pump after 5 s. According to S2 Table (this table shows the specific cleaning steps for different parts in a fine particle dose experiment, see supporting information), we used the rinsing solution to clean nebulizering container at each stage as test solution or its stock solution. High performance liquid chromatography was used to determine the content of sodium sivelestat in the rinsing solution of each part. According to the above method, different nebulizers were selected for detection, and each nebulizer was measured in parallel for 3 times. The results of rinsing solution of each part were imported into CopleyCitdas V3.10 software for calculation, and these parameters of mass median aerodynamic diameter (MMAD), fine particle dose (FPD), fine particle fraction (FPF) and geometric standard deviation (GSD) were obtained. The measuring results are shown in Table 1.

**Table 1:** Aerodynamic analysis data of different nebulizers ($\bar{x}$±s, n = 3).

Type of nubulizer		FPD(mg)	FPF%	NMAD (*μ*m)	GSD
Air compression	A	4.29±0.2^a^	49.73±2.0^a^	4.81±0.2^a^	2.17±0.1^a^
	B	4.91±0.4^a^	52.93±0.8^a^	4.61±0.1^a^	2.02±0.1^a^
Mesh	C	4.60±0.3^b^	62.05±1.3^b^	4.11±0.1^b^	1.79±0.1^b^
	D	5.04±0.4^b^	60.50±1.1^b^	4.17±0.2^b^	1.89±0.1^b^

When comparing data in the same column, different alphabets indicate statistically significant differences, this is *P*<*0*.*05*. And FPF = FPD/ emitted dose.

### Measurement of delivery rate and total emitted dose

The *in vitro* atomization time of nebulizer A, B, C and D was investigated, and 2 mL(A and B) or 1 mL(C and D) of 10 mg/mL self-developed formulation solution was added to the nebulizer, and no aerosol discharge was taken as the termination time of investigation. It was observed that type A and B nebulizers had no aerosol discharge in about 8 minutes, and type C and D nebulizers had no aerosol discharge in about 7 minutes, so the nebulization time was set at 10 minutes.

According to the fourth General Rule 0111 of ChP 2020 edition [23], the specific operation is as follows: 2 mL(A nad B) or 1 mL(C and D) of 10 mg/mL self-developed formulation solution were accurately measured and placed in a nebulizering cup, and the breath simulator was connected with the filter membrane, nebulizer and nebulizering cup device. The parameters of the breath simulator were set as adult mode (total volume 500 mL, breath rate 15 cycles/min, breath waveform sinusoidal, breath ratio 1:1). The breath simulator was started and the working time of the breath simulator was set as 60 s±1 s. Start breathing simulator and nebulizer at the same time. After 1 min of timing, close the nebulizer and collect the inhalation filter membrane. Replace the new filter membrane, continue to atomize for 9 min, and collect the inhalation filter membrane and exhalation filter membrane. Using diluent as solvent, each filter membrane and component were cleaned according to S3 Table (this table shows the specific cleaning steps for different parts in a fine particle dose experiment, see supporting information), filtered with 0.45 *μ*m microporous filter membrane, and used as test solution. The content of sodium sivelestat in the above washing liquor was determined by high performance liquid chromatography (HPLC). The delivery rate was the ratio of sodium sivelestat content in inhalant filter membrane to the nebulization time; the total emitted dose was the sum of sodium sivelestat in inhalant filter membrane; the total exhalation was the content of sodium sivelestat in exhalant filter membrane; the residual dose was the content of sodium sivelestat in nebulizing cup. The recovery is the percentage of the total measured amount (the sum of sodium sivelestat in each part) to the actual amount of sample used in the test. According to the above method, different nebulizers were selected for determination, and each nebulizer was measured in parallel for 3 times. The test results were shown in Table 2.

**Table 2:** Delivery data of different nebulizers ($\bar{x}$±*s*, n = 3).

Type of nubulizer		Delivery rate (*μ*g/s)	Total emitted dose(mg)	Total exhalant dose (mg)	Residual dose (mg)	Recovery%
Air Compression	A	27.11±0.5^a^	5.98±0.2^a^	1.18±0.6^a^	11.23±0.9^a^	100.30±1.3^a^
	B	29.89±1.1^a^	6.37±0.2^a^	1.14±0.3^a^	11.11±0.3^a^	97.55±1.4^a^
mesh	C	11.67±0.5^b^	7.91±0.4^b^	0.32±0.2^c^	2.47±0.1^b^	98.71±0.9^a^
	D	12.39±1.3^b^	7.67±0.3^b^	0.28±0.3^c^	1.91±0.2^b^	97.93±1.0^a^

When comparing data in the same column, different alphabets indicate statistically significant differences, this is *P*<*0*.*05*.

### Drug concentration detection and data process

The concentration of sodium sivelestat was determined using HPLC. Details of the sodium sivelestat assay method can be found in the supporting information. Data processing was carried out using both Microsoft Excel and SPSS 22.0 statistical software. Statistical analysis involved the application of univariate analysis of variance, and statistical significance was determined at *P<0.05.

### Ethics statement

The study was performed according to the international, national and institutional rules considering animal experiments, clinical studies and biodiversity rights. The study protocol was approved by Laboratory Animal Ethics Committee Jinan University.

### Experimental study on acute lung injury induced by aerosol inhalation of LPS in mice

For the nebulization procedure based on a whole body device, mice were placed in a sealed 15.5 × 23.2 × 31.6 cm plastic box which was connected directly to the nebulizer. Two small holes were made on the opposite side of the plastic box to allow aerosol flow through the box [24].

A total of 120 male ICR mice with a body weight ranging from 18 g to 22 g, all of SPF grade, were randomly divided into six groups based on their body weight. These groups included the normal control group, model control group, sodium sivelestat for injection control group, self-developed formulation solution groups with concentrations of 15 mg/mL, 10 mg/mL, and 3 mg/mL. Each group consisted of 20 mice. These mice were anesthetized with an intraperitoneal injection of 3% sodium pentobarbital (30 mg/kg) before giving an intratracheal instillation of LPS or sterile 0.9% saline for the first 3 days [25]. And then, with the exception of the normal control and model control groups, the self-developed formulation solution groups were subjected to a 15-minute atomization process. The control group received a 0.2 mL/10 g body weight injection via the tail vein, once daily for four consecutive days. The normal control and model control groups underwent a 15-minute atomization process using normal saline under the same conditions. To induce acute lung injury in the mice, two hours before administration on the fourth day, all groups except the normal control were exposed to LPS (20 mg/mL) for 20 minutes. After each instillation, we injected 3 mL air to lung with syringe for five times, and the rats were supine 2 minutes, then turned left, keeping the position 2 minutes, and then turned right, also keeping the position 2 minutes. At last, the rats turned left again, keeping the position until they awaked. All above we were done were to ensure all drug distribution in the lung.

Samples were collected after six hours of establishing the model. The following measurements and analyses were conducted: a. at the end of the experiment, eight mice from each group were dissected after being asphyxiated with carbon dioxide immediately, and their lung weights were used to calculate the lung index and inhibition rate. The left lung lobe was used to measure the contents of interleukin-6 (IL-6), tumor necrosis factor α (TNF-α), and α-protease inhibitor (α-AT). Statistical analysis was performed using a T-test to compare between groups; b. Another eight mice from each group had their alveolar lavage fluid collected to determine neutrophil elastase (NE) activity. This was done following the kit’s instructions, and a microplate reader was used for detection. Statistical analysis involved comparing results using a t-test between groups. The test results are provided in Table 3.

**Table 3:** Effects of aerosol inhalation of LPS on acute lung injury in mice.

Group	Actual administered dose (mg/kg/d)	Inhalation time(min)	specimen volume	lung index	Inhibition rate (%)	IL-6	TNF-α	α_1_-AT	NE activity
						(pg/mL)	(pg/mL)	(ng/mL)	(U/L)
Normal group	/	15	8	0.66±0.03	/	140.10±4.63	5041.01±426.72	5408.95±522.95	5.48±1.19
Model Control	/	15	8	0.81±0.02**	/	305.43±7.39**	6296.29±670.52**	6364.87±935.25*	15.93±10.03*
Sodium sivelestat for injection control	52.8	/	8	0.78±0.05	22.76	279.34±7.24^##^	5783.97±481.83	5866.08±520.40	9.99±6.73
15 mg/mL self-developed formulation	9.78	15	8	0.76±0.05^#^	33.62	266.20±6/08^##^	6314.42±536.60	6514.77±533.26	8.45±1.89
10 mg/mL self-developed formulation	6.37	15	8	0.76±0.04^##^	36.11	278.81±9.24^##^	6198.74±570.64	5232.93±434.85^##^	8.32±2.16
3 mg/mL self-developed formulation	2.08	15	8	0.75±0.05^##^	41.68	282.66±8.34^##^	5915.12±816.34	6155.30±818.54	8.50±5.12

Compared with normal control group

“**” stands for P<0.01

“*”stands for p<0.05: Compared with model control group

“^##^” stands for P<0.01

“^#^”stands for p<0.05.

## Results and discussion

### Formulation composition and freeze-drying

Sodium sivelestat requires a weakly alkaline environment for dissolution, but such conditions can lead to its degradation. Therefore, we opted for a freeze-dried preparation to maintain stability. To enhance the stability and formability of the lyophilized preparation, anhydrous ethanol was introduced into the formulation. We implemented a controlled stage heating program up to 40°C, which not only effectively controlled water content (resulting in a drying weight loss of less than 2.0%) but also contributed to the formability of our self-developed lyophilized preparation without leaving any ethanol residue. Additionally, besides the role of phosphate buffer salt as a pH regulator, phosphate buffer salt and sodium chloride together also serves as an osmotic pressure regulator and an excipient. In this formulation, referring to the literature [26, 27], we chose Tween 80 to solve the problem of uniformity of atomized droplets of this preparation. The results showed that the atomization uniformity of the preparation was good.

### Solution droplet size analysis

The resuls, as displayed in Fig 1, indicated that the self-made formulation solutions at concentrations of 15 mg/mL, 10 mg/mL, and 3 mg/mL, when subjected to atomization at a flow rate of 7.5 L/min, consistently maintained a median droplet size between 3.5 *μ*m and 3.8 *μ*m throughout the entire 30-minute period of atomized inhalation. The stability in aerosol droplet size over the 30-minute duration is a noteworthy finding.

### Spray modes

**Fig 2:**
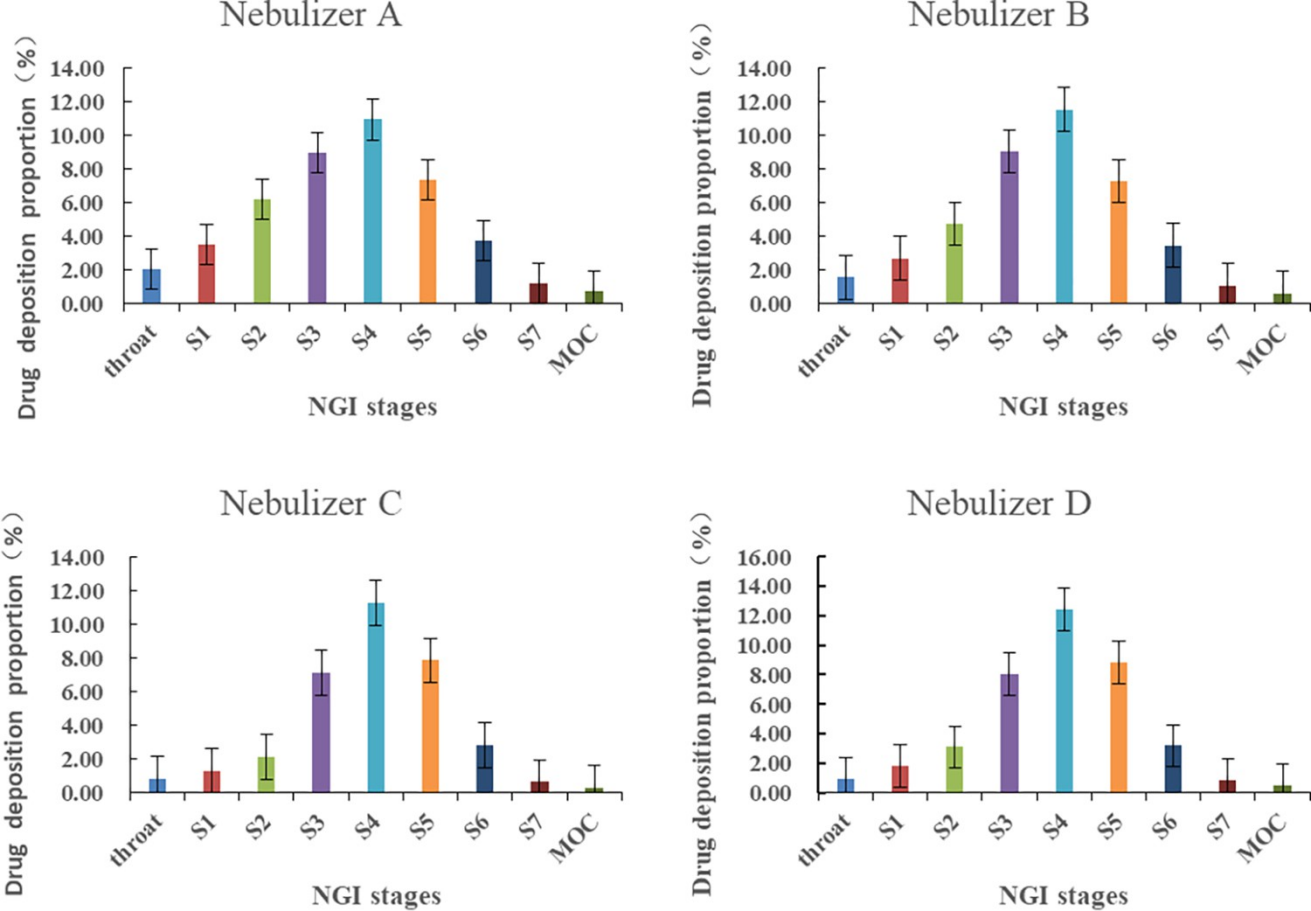
Deposition proportion at all levels of type A, type B, type C and type D nebulizers (cut-off sizes for apparatus at 15 L/min, S1(14.1 *μ*m)、S2(8.61 *μ*m)、S3(5.39 *μ*m)、S4(3.30 *μ*m)、S5(2.08 *μ*m)、S6(1.36 *μ*m)、S7(0.98 *μ*m)).

To assess the variation in drug delivery associated with different spray modes, we conducted research using two commonly employed nebulizers in the market, namely, the air compression nebulizer and the mesh nebulizer. The test solutions were analyzed according to the specified chromatographic conditions (for details, please refer to the supporting information), and the drug deposition amounts at stages 1–7 as well as stage MOC (Micro-orifice collector) for each nebulizer were calculated. The results are presented in Fig 2. Notably, when the same sample is subjected to different nebulizers, there are discernible differences in the amount of drug deposition at various stages of the NGI (Next-Generation Impactor), particularly between different nebulizer types. Among nebulizers A, B, C, and D, stage 4 exhibited the highest drug deposition, followed by stages 3 and 5, with stages 2 and 6 following. Of significance, the difference in drug deposition at stage 4 and stage 3 was smaller for air compression nebulizers A/B when compared to mesh nebulizers C/D. Additionally, the proportion distribution diagram of the two nebulizer types showed opposite orders for drug deposition at stage 2/6 and stage 3/5. These differences may result in variations in the inhaled dose, which, if not addressed, could impact the effectiveness of treatment.

### Aerodynamic particle size distribution

**Fig 3:**
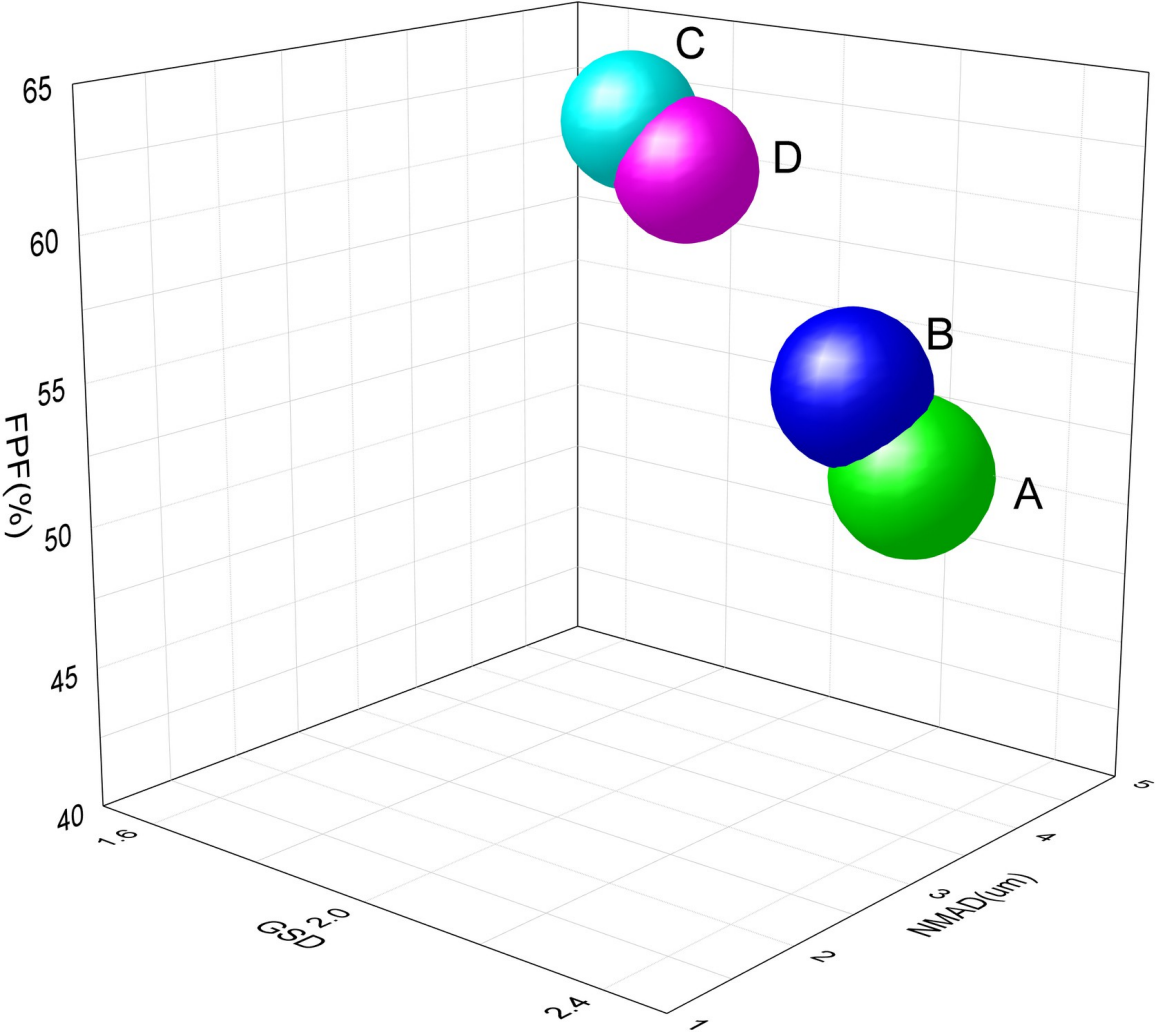
Three-dimensional bubble diagram of aerodynamic characteristics of fine particles in different nubulizers.

Chinese Pharmacopoeia, European Pharmacopoeia, and US Pharmacopoeia have provided various methods for detecting aerodynamic particle size distribution based on collision principles. In recent years, NGI (Next-Generation Impactor) has become a commonly used method [28]. Parameters such as MMAD (Mass Median Aerodynamic Diameter), FPD (Fine Particle Dose), FPF (Fine Particle Fraction), and GSD (Geometric Standard Deviation) are commonly used for evaluation. To calculate these parameters from the drug deposition at stages 1–7 and stage MOC, we utilized CITDA, specialized software for aerodynamic particle size distribution. As detailed in Table 1, the NGI measurements for mesh nebulizer C/D indicated a smaller MMPD and GSD compared to air compression nebulizer A/B. This suggests that the preparation’s aerosol droplet size is smaller and more uniform when using the mesh nebulizer, which is lined with literature [29, 30]. Furthermore, the FPF value for the mesh nebulizer was 10% higher than that for the air compression nebulizer, indicating the mesh nebulizer’s superior performance with our formulation. Three-dimensional diagrams depicting FPF, MMAD, and GSD were generated, as displayed in Fig 3. The differences between nebulizers A/B and C/D in different nebulization modes were statistically significant, as observed in the scattered data points in Fig 3. The clustered data points demonstrated that under the same nebulization mode, there were no statistically significant differences between nebulizers A and B, and similarly, between nebulizers C and D. Atomizer C exhibited the best aerodynamic results, with the smallest MMAD and GSD values, as well as the highest FPF values. This suggests that aerosol mass with the smallest MMAD and the narrowest particle size distribution (less than 5 *μ*m) was the largest under the influence of atomizer C.

### Delivery rate and total emitted dose

**Fig 4:**
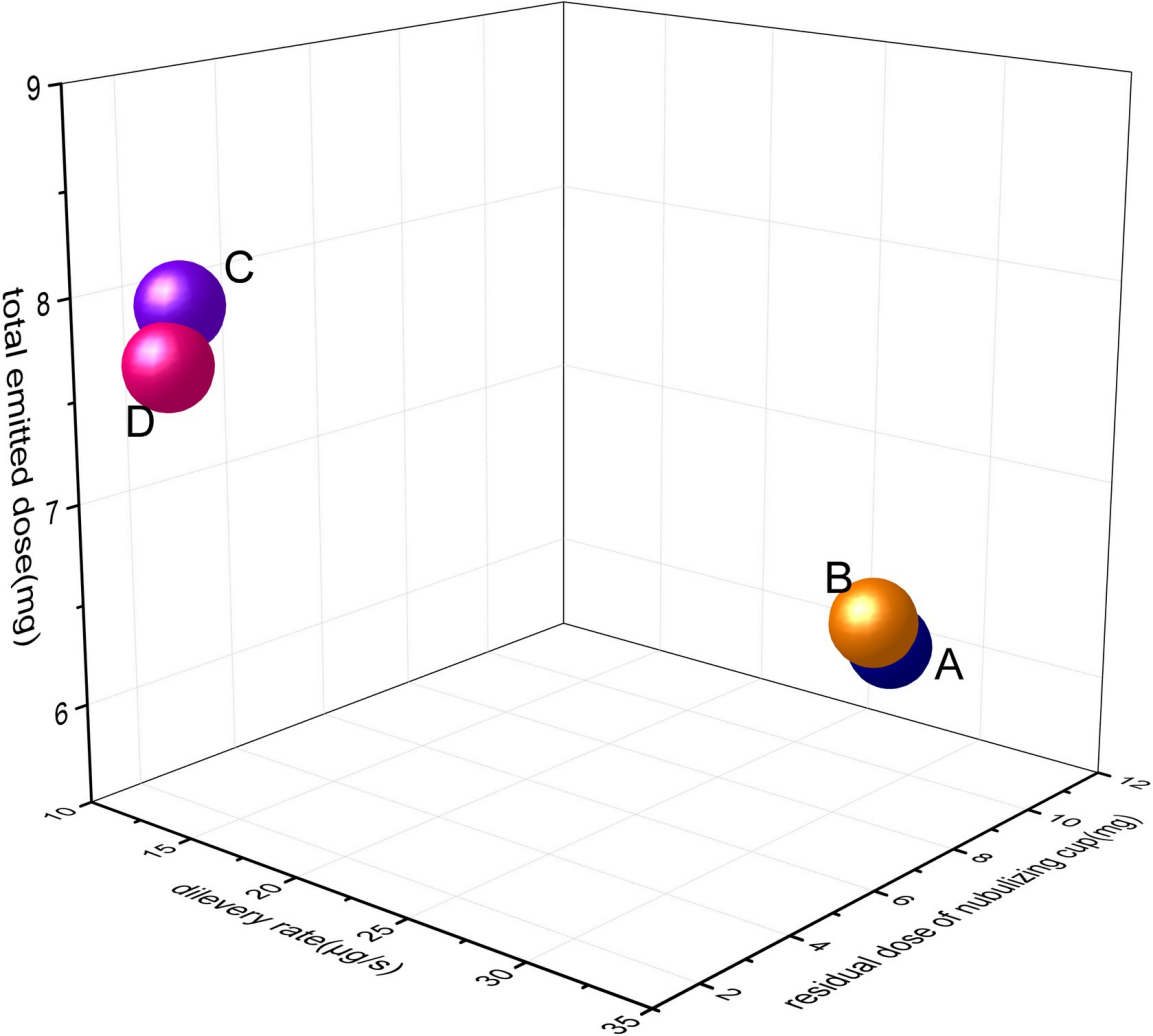
Three-dimensional bubble diagram of delivery characteristics of different nebulizers.

The results in Table 2 reveal significant differences in the delivery rate due to variations in the nebulizer’s power and the size of the nebulizing cup, especially when different spraying methods are employed. Nebulizer B exhibited the highest delivery rate, which was not statistically significant when compared to nebulizer A. Conversely, nebulizer C had the slowest delivery rate, with no statistically significant difference compared to nebulizer D. The data analysis for the total exhaled dose yielded statistically similar results to the delivery rate analysis. According to the total emitted dose, mesh nebulizers C/D outperformed air compression nebulizers A/B. The difference within the same type of nebulizer was not statistically significant, as indicated by the clustered points in Fig 4. However, significant differences were observed among different types of nebulizers, as shown by the scattered data points in Fig 4. These substantial differences between mesh nebulizers and air compression nebulizers are valuable for guiding actual inhalant administration. Furthermore, nebulizer D had the least residual dose in the nebulizing cup, and there was no statistically significant difference in the residual dose between nebulizer D and C. Nebulizer A exhibited the most residue, but there was no statistical significance when compared to nebulizer B. Noticeable differences in the residual dose were observed between mesh nebulizers C/D and air compression nebulizers A/B. This can be attributed to the larger volume of the nebulizer’s cup in the air compression nebulizer, which results in more residue left in the cup. In addition, the recoveries for nebulizers A, B, C, and D were all greater than 95%, indicating that the design of the nebulizer’s top cover effectively prevents nebulized droplets from being ejected during exhalation. The aforementioned washing methods successfully collected sodium sivelestat in all parts, and the filter membrane exhibited no adsorption effect on the drug.

### Comparison of acute lung injury induced by aerosol inhalation of LPS in mice

Table 3 presents the results, and it is evident that after acute lung injury induced by aerosol inhalation of LPS, the lung index in the model control group significantly exceeded that in the normal control group (P<0.01). However, the 15 mg/mL, 10 mg/mL, and 3 mg/mL self-developed formulation solutions markedly reduced the lung index, demonstrating significant differences when compared to the model control group (P<0.05, P<0.01). The inhibition rates of the lung index were 33.62%, 36.11%, and 41.68%, respectively.

Additionally, the levels of IL-6 and TNF-α in the lung tissue of mice in the model control group were significantly elevated compared to those in the normal control group following acute lung injury caused by aerosol inhalation of LPS (P<0.01). The tested self-developed formulation solutions at 15 mg/mL, 10 mg/mL, and 3 mg/mL significantly reduced the IL-6 content in the lung tissue of mice, exhibiting significant differences compared to the model control group (P<0.01). Moreover, the content of TNF-α in the lung tissue of mice in the 10 mg/mL self-developed formulation solution group was significantly lower than that in the normal control group (P<0.05).

Furthermore, the content of α1-AT in the lung tissue of mice in the model control group was significantly higher than that in the normal control group (P<0.05). In contrast, the content of α1-AT in the lung tissue of mice in the 10 mg/mL self-developed formulation solution group was significantly decreased, exhibiting significant differences compared to the model control group (P<0.01). NE activity in the alveolar lavage fluid of mice in the model control group was significantly higher than that in the normal control group (P<0.05). And then, the tested 15 mg/mL, 10 mg/mL, and 3 mg/mL self-developed formulations were able to inhibit NE activity in the alveolar lavage fluid of mice to some extent.

## Conclusions

This paper focused on the primary parameters of aerodynamic particle size distribution, including FPD, FPF, MMAD, and GSD, in fine particle mass experiments. Additionally, in the breath simulation experiment, the delivery rate and total emitted dose of commonly used nebulizers were evaluated. These in vitro atomization characteristics met the requirements for inhalation. The comparison results between two commonly used nebulizers indicated that mesh atomizers provided a smaller and more uniform particle size distribution. Moreover, following the induction of acute lung injury by aerosol inhalation of LPS, the treatment outcomes demonstrated that inhalation of sodium sivelestat at a lower concentration was equally effective as the injection of sodium sivelestat. Importantly, no other adverse effects of this preparation on the lungs of mice were observed during the experiment. Although all of these results collectively support the conclusion that the self-developed sodium sivelestat can be utilized as an aerosol inhalation drug, some shortages have also been existed. For example, physicochemical parameters such as viscosity and density of the formulation solution may also affect the atomization characteristics. Also, the final determination of the formulation largely needs to be evaluated through safety and stimulation testing. Next, we need to finalize the amount of active pharmaceutical ingredient in the formulation and conduct long-term studies on the stability of the prescription.

## Supporting information

S1 Fig The seperation chromtogram of sivelestat and propyl p-hydroxybenzoate. (TIF)

S1 Table Summary of the parameters of each nebulizer used. (DOCX)

S2 Table Specific cleaning steps for different parts in a fine particle dose experiment. (DOCX)

S3 Table Specific cleaning steps of different modules in a breath simulator experiment. (DOCX)

S1 File Method validation of content of sodium sivelestat. (DOCX)

S2 File (DOCX)

S1 Raw data (XLSX)
