# Supplementary figures and images for: In vitro atomization analysis and evaluation of inhalable sodium sivelestat formulations
Source: PLoS One. 2024 Sep 20;19(9):e0309721. doi: 10.1371/journal.pone.0309721 (PMC11414907; doi:10.1371/journal.pone.0309721)

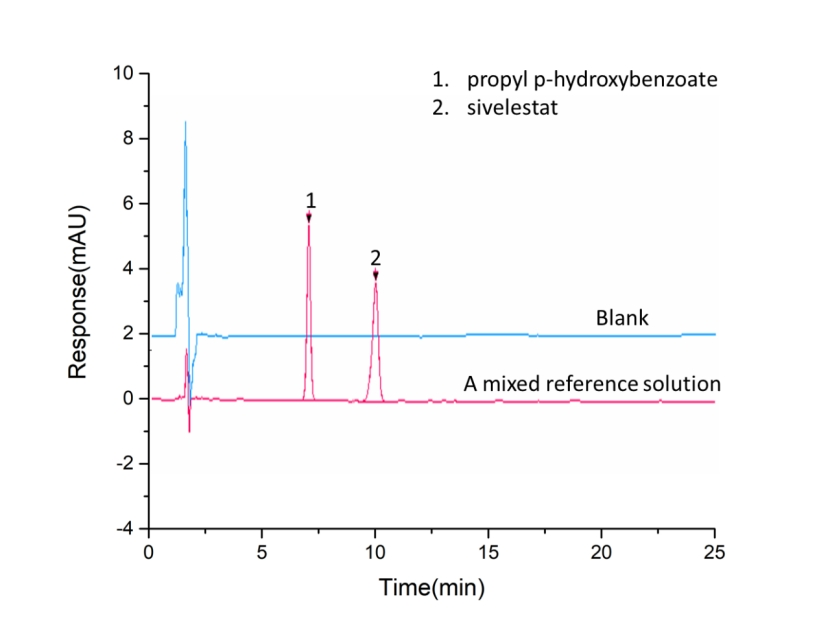

Supplement: S1 Fig — (TIF) [file pone.0309721.s001.tif]
