# Supplementary material for: In vitro atomization analysis and evaluation of inhalable sodium sivelestat formulations
Source: PLoS One. 2024 Sep 20;19(9):e0309721. doi: 10.1371/journal.pone.0309721 (PMC11414907; doi:10.1371/journal.pone.0309721)
Supplement: S1 Table — (DOCX) [file pone.0309721.s002.docx]

S1_table. Summary of the parameters of each nebulizer used

| Nebulizers | Volume of nebulizer`s cup( mL) | Median sizeum(μm) | Atomization rate(mL/min) | Gas flow (L/min) |
| --- | --- | --- | --- | --- |
| 403H compressed air nebulizer (A) | 3 | 2.54，The proportion of effective particles below 5 μm＞74% | ≥0.2 | 10 |
| 085 compressed air nebulizer (B) | 4 | 3.5, The proportion of effective particles below 5 μm＞67% | 0.5 | 10.9 |
| Air Pro Ⅷ Mesh nebulizer (C) | 10 | 2.5，The proportion of effective particles below 5 μm＞68% | 0.15 -0.90 | / |
| M105 Mesh nebulizer(D) | 8 | 3.7，The proportion of effective particles below 5 μm＞60% | ≥0.2 | / |
