# Supplementary material for: In vitro atomization analysis and evaluation of inhalable sodium sivelestat formulations
Source: PLoS One. 2024 Sep 20;19(9):e0309721. doi: 10.1371/journal.pone.0309721 (PMC11414907; doi:10.1371/journal.pone.0309721)
Supplement: S2 Table — (DOCX) [file pone.0309721.s003.docx]

S2_table. Specific cleaning steps for different parts in a fine particle dose experiment

| Part | Preparation of test solutions |
| --- | --- |
| Nebulizing Cup | According to the method of small amount and multiple times, clean the nebulizing cup with solvent, transfer the lotion to 50 mL volumetric flask, and quantitatively dilute with solvent to the scale, then obtain stock solution. Take 1 mL this stock solution and 3 mL internal standard solution and put them into 10 mL volumetric flask with solvent, shake well, filter, and obtain. |
| Stage1、Stage6 | According to the method of small amount and multiple times, clean the nebulizing cup with solvent, and transfer the lotion to 20 mL volumetric flask, each volumetric flask is added with 6 mL of internal standard solution, fixed volume with solvent to the scale, shake well, filter, and obtain. |
| throat、Stage7、Micro-orifice collector (MOC) | According to the method of small amount and multiple times, each part was cleaned with solvent, and the lotion was transferred to 10 mL volumetric flask, each volumetric flask was added with 3 mL of internal standard solution in advance, fixed volume with solvent to the scale, shake well, filter, and obtain. |
| Stage2 | According to the method of small amount and multiple times, each part was cleaned with solvent, and the lotion was transferred to 25 mL volumetric flask, each bottle was added with 7.5 mL internal standard solution in advance, fixed volume with solvent to the scale, shake well, filter, and obtain. |
| Stage3、Stage4、Stage5 | According to the method of small amount and multiple times, clean the nebulizing cup with solvent, transfer the lotion to a 5 mL volumetric flask, quantitatively dilute with solvent to the scale, and obtain stock solution. Take 1 mL this stock solution and 3 mL internal standard solution and put them into a 10 mL volumetric flask with solvent, shake well, filter, and obtain. |
