# Supplementary material for: In vitro atomization analysis and evaluation of inhalable sodium sivelestat formulations
Source: PLoS One. 2024 Sep 20;19(9):e0309721. doi: 10.1371/journal.pone.0309721 (PMC11414907; doi:10.1371/journal.pone.0309721)
Supplement: S3 Table — (DOCX) [file pone.0309721.s004.docx]

S3_table. Specific cleaning steps of different modules in a breath simulator experiment

| Parts | Preparation of test solutions |
| --- | --- |
| Nebulizing cup | Use a small amount for several times to clean the residual liquid in the nubulized cup with diluent, and transfer the lotion into a 50 mL volumetric flask. Take 2 mL this solution and 1 mL internal standard solution, and place them in a 10ml volumetric bottle, and dilute it with diluent to the scale. |
| Inhalation filter membrane | Place in a 50 mL centrifuge tube, add 1-2 mL diluent solution, swirl for 1 min, place filter paper in a 20 mL syringe, press a small amount of times, clean the adsorbed drug solution in the filter membrane with the diluent, and transfer the lotion into a 20ml volumetric flask, dilute it with the diluent to the scale, shake well; Take 2 mL of the solution and 1 mL of the internal standard solution into a 10 mL measuring bottle, dilute it with diluent to the scale, shake well. |
| Inhalation filter membrane | Place in a 50 mL centrifuge tube, add 1-2 mL diluent solution, swirl for 1 min, place filter paper in a 20 mL syringe, press a small amount of times, clean the adsorbed drug solution in the filter membrane with the diluent, and transfer the lotion into a 20ml volumetric flask, dilute it with the diluent to the scale, shake well; Take 2 mL of the solution and 1 mL of the internal standard solution into a 10 mL measuring bottle, dilute it with diluent to the scale, shake well. |
| Exhalation filter membrane | Place in a 50 mL centrifuge tube, add 1-2 mL diluent solution, swirl for 1 min, place filter paper in a 20 mL syringe, press a small amount of times, clean the adsorbed drug solution in the filter membrane with the diluent, and transfer the lotion into a 20ml measuring bottle, dilute it with the diluent to the scale, shake well; Take 2 mL of the solution and 1 mL of the internal standard solution into a 10 mL measuring bottle, dilute it with diluent to the scale, shake well. |
| Other connecting device | The residual liquid in the nubulizing cup was cleaned with diluent several times, and the lotion was transferred to a 50 mL volumetric flask. 5 mL solution was placed in 1 mL internal standard solution into a 10 mL volumetric bottle, and diluted to the scale with diluent. |
