# Supplementary material for: In vitro atomization analysis and evaluation of inhalable sodium sivelestat formulations
Source: PLoS One. 2024 Sep 20;19(9):e0309721. doi: 10.1371/journal.pone.0309721 (PMC11414907; doi:10.1371/journal.pone.0309721)
Supplement: S2 File — (DOCX) [file pone.0309721.s006.docx]

**Supporting Information**

**In vitro atomization analysis and evaluation of inhalable sodium sivelestat formulations**

Rangdong Liu*^1,2,3^, Aifang He^3^, Yan Xu^3^, Yisheng Zhou^3^, Hui Cao^2^

1. Zhuhai College of Science and Technology, Zhuhai, 519040, China;
2. Jinan University, College of Pharmcy, Guangzhou, 510660, China;
3. Increase pharma (Hengqin) Institute Co. LTD, Zhuhai, 519031, China.

**^*^Corresponding author**

1. mail: rangdongliu@163.com

Affiliation: 1. Zhuhai College of Science and Technology, Zhuhai, 519040, China; 2. Jinan University, College of Pharmcy, Guangzhou, China; 3. Increase pharma (Hengqin) Institute Co. LTD, Zhuhai, China

**1. Assay for sodium sivelestat**

*1.1. Chromatographic condition*

Welch Ultimate® XB-C18 was used as a column (150 mm×4.6 mm, 5 *μ*m). Acetonitrile-phosphate buffer (5:4, v/v) was used as mobile phase. The detection wavelength was 240 nm. The injuction volume was 10 *μ*L. The content of sivelestat in samples was calculated by internal standard method. The number of theoretical plates calculated by the peak of sivelestat should be not less than 5000, and the resolution between the main peak of sivelestat and the internal standard peak of propyl hydroxybenzoate was not less than 5.0. The chromatogram is shown in S-Fig.1.

*1.2. Solution Preparation*

*1.2.1. Preparation of standard solution*

Sivelestat stock solution: weigh 25.55 mg sivelestat into 25 mL volumetric flask, add acetonitrile to dissolve it and shake well.

Sodium propyl p-hydroxybenzoate stock solution: weigh 42.39 mg sodium propyl p-hydroxybenzoate into 100 mL volumetric flask, add acetonitrile to dissolve it and shake well.

1 mL sivelestat stock solution and 1 mL stock solution of sodium propyl p-hydroxybenzoate were accurately added to a 100 mL volumetric flask, and then use 50% acetonitrile to dilute this flask to the scale, mixing them well.

*1.2.2. Preparation of test solution*

7.5mL water was added to a celine bottle containing homemade sodium sivelestat lyophilized powder, shaking well and labling it as TS. Accurately remove 1 mL TS to a 10 mL volumetric flask, and then add 1 mL stock solution of propyl sodium p-hydroxybenzoate to this one, and dilute it with 50% acetonitrile to the scale, shake well, filter it with 0.45 *μ*m microporous filter membrane, take the filtrate to injection.

*1.3. Experimental contents and Results*

*1.3.1. The establishment of a standard curve*

Precise removal of different volumes of sivelestat stock solution and sodium propyl p-hydroxybenzoate stock solution, adding 50% ACN dilution to the scale, A series of reference solutions were prepared with the mass concentration of sivelestat at 0.9954, 4.977, 9.954, 19.91, 99.54 *μ*g/mL (the internal standard solution concentration in different linear solutions is the same as 4.208 *μ*g/mL). According to the chromatographic conditions under "2.1.1", the standard curve was plotted with mass concentration ratio as horizontal coordinate and peak area ratio as vertical coordinate. The standard curve Y=0.8378X-0.0458 (r=1.0000) was obtained, and the linear range was 0.9954~99.54 *μ*g/mL.

*1.3.2. Precision*

The same test solution was injected for 6 times according to the chromatographic conditions of "2.1.1". Relative standard variation (RSD) of the ratio of peak area between the main peak of sivelestat and the internal standard peak of propyl p-hydroxybenzoate was less than 1.0%, indicating good precision of the instrument.

*1.3.3. Repeatability*

The same batch of sodium sivelestat lyophilized powder was prepared according to the method of "2.1.3", and 6 parallel solutions were prepared according to the chromatogenic conditions under "2.1.1". The result showed that the RSD of the ratio of peak area of sivelestat to the internal standard peak of propyl p-hydroxybenzoate was less than 1.0%. The resolution between the main peak of sivelestat and the internal standard peak of propyl p-hydroxybenzoate was greater than 5.0, indicating good repeatability of the method.

*1.3.4. Solution stability test*

The same batch of sodium sivelestat lyophilized powder was used to prepare the test solution according to "2.1.3" method. The solution was determined at 0, 2, 4, 8, 16 and 24 h after preparation on the basis of "2.1.1". The results showed that the peak-area RSD of sivelestat was less than 2.0%, indicating that the solution was stable within 24 h.

*1.3.5. Precision*

The lyophilized powder of sodium sivelestat was accurately weighed, and a certain amount of sivelestat standard substance was added. The test solution was prepared according to the method under "2.1.3", and 6 parallel samples were determined according to the chromatographic conditions under "2.1.1", and the recovery was calculated by internal standard method. The results showed that the average recovery was 99.68% and RSD was 0.9%, indicating good accuracy of the method.

**2. Studying for ringsing solution of atomization experiment**

Ethanol and phosphate can increase the solubility of sodium sivelestat because of its low water solubility. In addition, sodium sivelestat has poor durability to pH, for instance, excessive acid conditions will precipitate it, and excessive alkali conditions would increase its hydrolytic products. As a result of the above, we chose 0.01M sodium dihydrogen phosphate solution with pH 7.9 of 20% ethanol as a washing liquor for this nebulization experiment. The experimental result indicated that the selected washing solution can meet the test requirement, and the recovery rate was 99.3%.

**3. S_Figure and S_table**


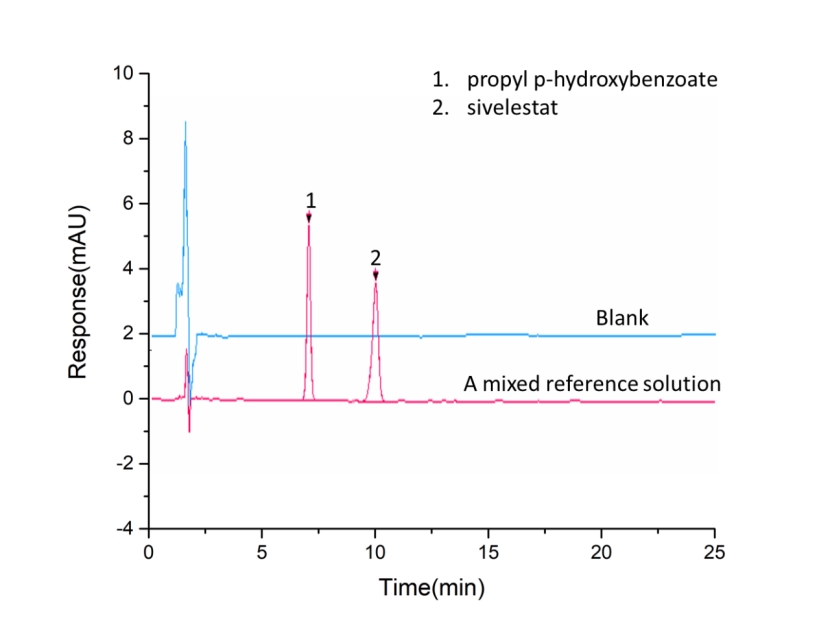


S1_Fig. The seperation chromtogram of sivelestat and propyl p-hydroxybenzoate

S1_table. Summary of the parameters of each nebulizer used

| Nebulizers | Volume of nebulizer`s cup( mL) | Median sizeum(μm) | Atomization rate(mL/min) | Gas flow (L/min) |
| --- | --- | --- | --- | --- |
| 403H compressed air nebulizer (A) | 3 | 2.54，The proportion of effective particles below 5 μm＞74% | ≥0.2 | 10 |
| 085 compressed air nebulizer (B) | 4 | 3.5, The proportion of effective particles below 5 μm＞67% | 0.5 | 10.9 |
| Air Pro Ⅷ Mesh nebulizer (C) | 10 | 2.5，The proportion of effective particles below 5 μm＞68% | 0.15 -0.90 | / |
| M105 Mesh nebulizer(D) | 8 | 3.7，The proportion of effective particles below 5 μm＞60% | ≥0.2 | / |

S2_table. Specific cleaning steps for different parts in a fine particle dose experiment

| Part | Preparation of test solutions |
| --- | --- |
| Nebulizing Cup | According to the method of small amount and multiple times, clean the nebulizing cup with solvent, transfer the lotion to 50 mL volumetric flask, and quantitatively dilute with solvent to the scale, then obtain stock solution. Take 1 mL this stock solution and 3 mL internal standard solution and put them into 10 mL volumetric flask with solvent, shake well, filter, and obtain. |
| Stage1、Stage6 | According to the method of small amount and multiple times, clean the nebulizing cup with solvent, and transfer the lotion to 20 mL volumetric flask, each volumetric flask is added with 6 mL of internal standard solution, fixed volume with solvent to the scale, shake well, filter, and obtain. |
| throat、Stage7、Micro-orifice collector (MOC) | According to the method of small amount and multiple times, each part was cleaned with solvent, and the lotion was transferred to 10 mL volumetric flask, each volumetric flask was added with 3 mL of internal standard solution in advance, fixed volume with solvent to the scale, shake well, filter, and obtain. |
| Stage2 | According to the method of small amount and multiple times, each part was cleaned with solvent, and the lotion was transferred to 25 mL volumetric flask, each bottle was added with 7.5 mL internal standard solution in advance, fixed volume with solvent to the scale, shake well, filter, and obtain. |
| Stage3、Stage4、Stage5 | According to the method of small amount and multiple times, clean the nebulizing cup with solvent, transfer the lotion to a 5 mL volumetric flask, quantitatively dilute with solvent to the scale, and obtain stock solution. Take 1 mL this stock solution and 3 mL internal standard solution and put them into a 10 mL volumetric flask with solvent, shake well, filter, and obtain. |

S3_table. Specific cleaning steps of different modules in a breath simulator experiment

| Parts | Preparation of test solutions |
| --- | --- |
| Nebulizing cup | Use a small amount for several times to clean the residual liquid in the nubulized cup with diluent, and transfer the lotion into a 50 mL volumetric flask. Take 2 mL this solution and 1 mL internal standard solution, and place them in a 10ml volumetric bottle, and dilute it with diluent to the scale. |
| Inhalation filter membrane | Place in a 50 mL centrifuge tube, add 1-2 mL diluent solution, swirl for 1 min, place filter paper in a 20 mL syringe, press a small amount of times, clean the adsorbed drug solution in the filter membrane with the diluent, and transfer the lotion into a 20ml volumetric flask, dilute it with the diluent to the scale, shake well; Take 2 mL of the solution and 1 mL of the internal standard solution into a 10 mL measuring bottle, dilute it with diluent to the scale, shake well. |
| Inhalation filter membrane | Place in a 50 mL centrifuge tube, add 1-2 mL diluent solution, swirl for 1 min, place filter paper in a 20 mL syringe, press a small amount of times, clean the adsorbed drug solution in the filter membrane with the diluent, and transfer the lotion into a 20ml volumetric flask, dilute it with the diluent to the scale, shake well; Take 2 mL of the solution and 1 mL of the internal standard solution into a 10 mL measuring bottle, dilute it with diluent to the scale, shake well. |
| Exhalation filter membrane | Place in a 50 mL centrifuge tube, add 1-2 mL diluent solution, swirl for 1 min, place filter paper in a 20 mL syringe, press a small amount of times, clean the adsorbed drug solution in the filter membrane with the diluent, and transfer the lotion into a 20ml measuring bottle, dilute it with the diluent to the scale, shake well; Take 2 mL of the solution and 1 mL of the internal standard solution into a 10 mL measuring bottle, dilute it with diluent to the scale, shake well. |
| Other connecting device | The residual liquid in the nubulizing cup was cleaned with diluent several times, and the lotion was transferred to a 50 mL volumetric flask. 5 mL solution was placed in 1 mL internal standard solution into a 10 mL volumetric bottle, and diluted to the scale with diluent. |
